# Dysregulation of Signaling Pathways Due to Differentially Expressed Genes From the B-Cell Transcriptomes of Systemic Lupus Erythematosus Patients – A Bioinformatics Approach

**DOI:** 10.3389/fbioe.2020.00276

**Published:** 2020-04-30

**Authors:** S. Udhaya Kumar, D. Thirumal Kumar, R. Siva, C. George Priya Doss, Salma Younes, Nadin Younes, Mariem Sidenna, Hatem Zayed

**Affiliations:** ^1^School of Biosciences and Technology, Vellore Institute of Technology, Vellore, India; ^2^Department of Biomedical Sciences, College of Health and Sciences, QU Health, Qatar University, Doha, Qatar

**Keywords:** systemic lupus erythematosus, protein–protein interactions, Metacore, microarray and bioinformatics, expression profiling data, biomarkers, functional enrichment analysis

## Abstract

Systemic lupus erythematosus (SLE) is an autoimmune inflammatory disorder that is clinically complex and has increased production of autoantibodies. Via emerging technologies, researchers have identified genetic variants, expression profiling of genes, animal models, and epigenetic findings that have paved the way for a better understanding of the molecular and genetic mechanisms of SLE. Our current study aimed to illustrate the essential genes and molecular pathways that are potentially involved in the pathogenesis of SLE. This study incorporates the gene expression profiling data of the microarray dataset GSE30153 from the Gene Expression Omnibus (GEO) database, and differentially expressed genes (DEGs) between the B-cell transcriptomes of SLE patients and healthy controls were screened using the GEO2R web tool. The identified DEGs were subjected to STRING analysis and Cytoscape to explore the protein–protein interaction (PPI) networks between them. The MCODE (Molecular Complex Detection) plugin of Cytoscape was used to screen the cluster subnetworks that are highly interlinked between the DEGs. Subsequently, the clustered DEGs were subjected to functional annotation with ClueGO/CluePedia to identify the significant pathways that were enriched. For integrative analysis, we used GeneGo Metacore^TM^, a Cortellis Solution software, to exhibit the Gene Ontology (GO) and enriched pathways between the datasets. Our study identified 4 upregulated and 13 downregulated genes. Analysis of GO and functional enrichment using ClueGO revealed the pathways that were statistically significant, including pathways involving T-cell costimulation, lymphocyte costimulation, negative regulation of vascular permeability, and B-cell receptor signaling. The DEGs were mainly enriched in metabolic networks such as the phosphatidylinositol-3,4,5-triphosphate pathway and the carnitine pathway. Additionally, potentially enriched pathways, such as the signaling pathways induced by oxidative stress and reactive oxygen species (ROS), chemotaxis and lysophosphatidic acid signaling induced via G protein-coupled receptors (GPCRs), and the androgen receptor activation pathway, were identified from the DEGs that were mainly associated with the immune system. Four genes (*EGR1*, *CD38*, *CAV1*, and *AKT1*) were identified to be strongly associated with SLE. Our integrative analysis using a multitude of bioinformatics tools might promote an understanding of the dysregulated pathways that are associated with SLE development and progression. The four DEGs in SLE patients might shed light on the pathogenesis of SLE and might serve as potential biomarkers in early diagnosis and as therapeutic targets for SLE.

## Introduction

Systemic lupus erythematosus (SLE), also known as lupus, is a rare systemic autoimmune disease that mostly affects middle-aged women, mainly of Asian, African, American, and Hispanic origin (Costa-Reis and Sullivan, 2013; Cui et al., 2013; Gurevitz et al., 2013). SLE affects an estimated 5 million people across the world, with an incidence of 1–10 per 100,000 person-years (Pons-Estel et al., 2010). SLE is characterized by a wide range of different autoantibodies, deposition of immune complexes, and immune system infiltration and inflammation within damaged organs. SLE autoantibodies invade the patient’s kidneys, heart, skin, joints, and brain, leading to various typical clinical symptoms. The most common clinical symptoms of lupus are rash, arthritis, and fatigue. Severe complications of SLE lead to nephritis, anemia, neurological symptoms, and thrombocytopenia, eventually leading to severe morbidity and mortality.

SLE is characterized by its clinical heterogeneity, with a wide range of clinical manifestations reflecting its complex etiopathogenesis (Tan et al., 1982). The clinical heterogeneity of SLE highlights the contribution of genetic and environmental factors to the susceptibility to the disease (Prokunina and Alarcon-Riquelme, 2004; Harley et al., 2009; Yang and Lau, 2015; Dang et al., 2016; Wang et al., 2017). To date, the reason for phenotypic variation in SLE is unknown. Understanding the molecular mechanisms behind the pathogenesis of SLE phenotypes could help in developing more efficient therapeutic approaches and preventive strategies.

With the extensive use of gene detection methods, high-throughput sequencing and extensive microarray data profiling studies on SLE have been conducted, and several differentially expressed genes (DEGs) and cellular pathways in SLE have been identified (Borrebaeck et al., 2014; Zhu et al., 2015). Nevertheless, until now, no particular gene has been recognized to act as a potential marker for the diagnosis of SLE. In addition, a large amount of data obtained from microarray technology and high-throughput sequencing have not been fully used. Ducreux et al. (2016) collected blood samples from SLE patients and healthy volunteers to identify differentially expressed genes (Ducreux et al., 2016). However, the interactions among differentially expressed genes and key genes involved in the signaling pathways of SLE remain to be elucidated. In addition, previous studies of genetic factors primarily focused on single genes; nevertheless, interactions among multiple genes may result in the multisystem invasion characteristics observed in SLE (Smith et al., 2017). Remarkably, studies have shown that disease−associated gene expression networks have a potential role in the immune response, which highlights their mechanism and therapeutic value for SLE (Deng and Tsao, 2010; Bentham et al., 2015).

Integrating and reanalyzing the data using bioinformatics methods may help in identifying gene regulatory pathways, essential genes, and their associated networks in SLE disease, which can provide new and valuable ideas for understanding the molecular mechanisms and identifying reliable diagnostic and therapeutic targets of SLE. Therefore, in this study, we first conducted a comprehensive collection of genes associated with SLE from the GEO dataset with ID GSE30153. Then, we performed a bioinformatics analysis of these genes with the MCODE (Molecular Complex Detection), GeneGo, and ClueGO tools. To further explore the pathogenesis of SLE in a more specific manner, functions and pathways identified by the modules were used to indicate the biological processes and biochemical pathways related to the immune system. Finally, the genes potentially associated with arthritis, pleurisy, and myocarditis, which are the common complications of SLE, were compared with SLE-related genes to identify common genes that participated in the development of SLE. To interpret the biological relevance of these changes in gene expression, we analyzed the microarray data via an integrated bioinformatic analysis expanding on traditional microarray analysis methods, namely, Gene Ontology (GO) and pathway analysis, thereby allowing the construction of interaction networks that might identify novel prognostic markers and therapeutic targets.

## Materials and Methods

### Acquisition of Array Data and Processing

Gene expression profiling data from microarray array analysis of the GSE30153 dataset were downloaded from the NCBI GEO database (Gene Expression Omnibus database)^1^. The database accommodates gene expression datasets from a variety of experiments, such as DNA-seq, ChIPs, RNA-seq, microarray, and high-throughput hybridization array (Edgar et al., 2002; Barrett et al., 2013). GSE30153 contains 26 samples, including 17 patients with SLE and 9 healthy controls of human sorted B-cells obtained by using the platform GPL570 (HG-U133_Plus_2) Affymetrix Human Genome U133 Plus 2.0 Array (Garaud et al., 2011). The downloaded gene expression profiling data are freely available in the public database, and there were no human or animal experiments conducted by any of the authors in this study.

### Preprocessing of Data and DEG Identification

Using the robust multiarray standard model, the initial information from the dataset was subjected to quantile normalization, background correction, and log transition (Irizarry et al., 2003). Preprocessing included changing to gene symbols from probe IDs using the Gene ID converter from Entrez (Alibés et al., 2007). The statistical online tool GEO2R uses the R/Bioconductor, and limma package v3.26.8 was used to screen the raw gene expression data (Smyth, 2005; Barrett et al., 2013; Ritchie et al., 2015). We performed a Benjamini–Hochberg test (to determine the false discovery rate) and *T*-tests to compute the false discovery rate (FDR) and *p*-values to identify the DEGs between SLE patients and healthy control human sorted B-cells (Benjamini and Hochberg, 1995; Aubert et al., 2004). We set the primary criteria of | log (2 fold change) | > 1 and *p* < 0.05 to obtain significant DEGs from the dataset, whereas cutoffs of log2FC ≥ 1 and log2FC ≤ −1 were used to denote upregulated and downregulated DEGs, respectively. For high-throughput sequencing, a logarithm to base 2 is widely used and in the initial scaling, the doubling is equivalent to a log2FC of 1 (Love et al., 2014). A volcano plot was constructed using a web-based tool^2^. The resulting DEGs were used for further analysis.

### Constructing PPI Networks

To assess the relationships between the DEGs from the GSE30153 dataset, we constructed a protein–protein interaction (PPI) network by using Search Tool for the Retrieval of Interacting Genes (STRING v11.0)^3^ (Szklarczyk et al., 2017, 2019). The cutoff criterion was set to a high confident interaction score of ≥0.7 to eliminate inconsistent PPIs from the dataset. We then incorporated the results from the STRING database into Cytoscape software (v3.7.2)^4^ to envisage the PPIs within the statistically relevant DEGs (Shannon et al., 2003). The MCODE plugin from Cytoscape was utilized to identify the interconnected regions or clusters from the PPI network. The cluster finding parameters were adopted, such as a degree cutoff of 2, a node score cutoff of 0.2, a kappa score (K-core) of 5, and a max depth of 100, which limits the cluster size for coexpressing networks (Bader and Hogue, 2003). The top clusters from MCODE were subjected to ClueGO v2.5.5/CluePedia v1.5.5 analysis to obtain comprehensive GO and pathway results from the PPI network. ClueGO combines GO and pathway analyses from KEGG and BioCarta and provides a fundamentally structured GO or pathway network from the PPI network (Bindea et al., 2009).

### Metacore GeneGo Analysis of DEGs

Metacore, a Cortellis Solution software (Clarivate Analytics, London, United Kingdom)^5^, was used to perform curated pathway enrichment analysis and GO analysis. GeneGo facilitates the rapid assessment of metabolic pathways, protein biological networks, and pathway maps from high-throughput experimental data (MetaCoreLogin | Clarivate Analytics). Based on a significance threshold of *p* < 0.05, a pictorial representation of the molecular interactions of DEGs from the study groups is generated. Determination of a hypergeometric *p*-value enables the estimation of the chance that an intersection between DEGs and ontological elements is random. An FDR < 0.05 was used as a criterion to calculate if statistically significant DEGs constituted a processor pathway.

## Results

### Identification of DEGs From the Dataset

**FIGURE 1:**
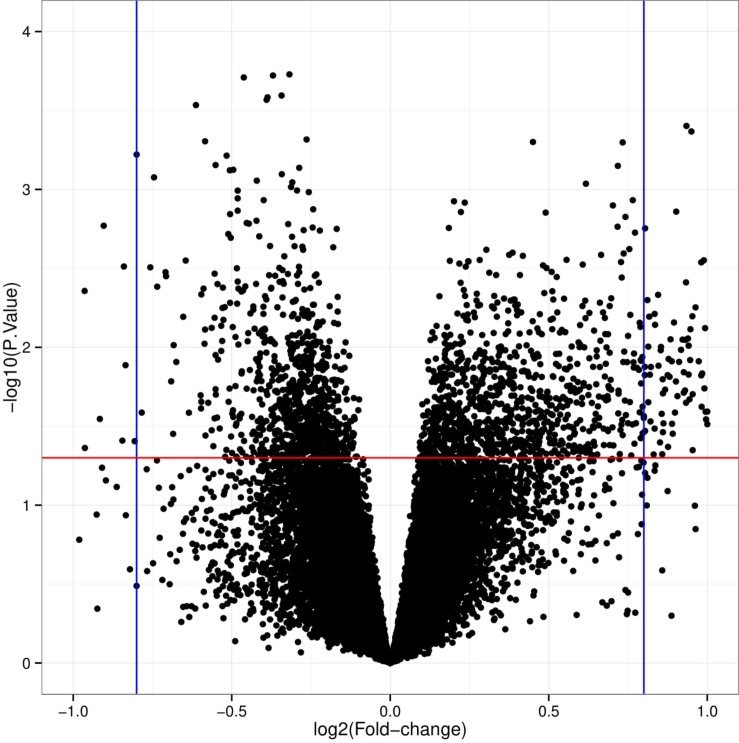
Pictorial representation of volcano plot for differentially expressed genes (DEGs) in systemic lupus erythematosus (SLE) compared to controls from the GSE30153 dataset. The X-axis represents Log2FC, large magnitude fold changes; Y-axis represents −log10 of a *p*-value, high statistical significance. Each black dot represents one gene. Black dots above red and beside blue line (left-sided and right-sided) are log2FC ≥ 1 and *p*-value <0.05, representing SLE related DEGs.

Our study contained the gene expression profiles of the GSE30153 dataset from the GEO database, which were submitted by Garaud et al. (2011) based on analysis with the GPL570 platform (Affymetrix Human Genome U133 Plus 2.0 Array) (Garaud et al., 2011). The dataset encompasses 26 samples, including 17 patients with SLE and 9 healthy controls (Table 1). By utilizing the GEO2R online tool, we obtained the differentially expressed genes (DEGs) from the GSE30153 dataset by comparing the SLE samples with control samples. By calculating *p*-values and | log2FC | values, the top 250 DEGs were identified. A volcano plot was constructed using the Rstudio web server ShinyVolcanoPlot to identify DEGs by comparing the SLE and control groups from the dataset. The volcano plot in Figure 1 depicts all the DEGs with a log2FC against the – log10 (*p*-value) between the two groups. With cutoffs of *p* < 0.05 and log2FC ≥ 1.0 or ≤−1, we found 4 and 13 genes that were upregulated and downregulated, respectively, between the two groups (Table 2). The genes that were differentially expressed between the two groups are shown in Supplementary Table S1.

**TABLE 1:** The primary characteristics of 26 studies in GSE30153 procured from the Gene Omnibus Expression database.

Group	Accession	Title	Organism	Disease state	Tissue	Cell type
Patient	GSM746726	Patient 1	Homo sapiens	Systemic lupus erythematosus (SLE)	Blood	Human sorted B cell
	GSM746727	Patient 2	Homo sapiens	Systemic lupus erythematosus (SLE)	Blood	Human sorted B cell
	GSM746728	Patient 3	Homo sapiens	Systemic lupus erythematosus (SLE)	Blood	Human sorted B cell
	GSM746729	Patient 4	Homo sapiens	Systemic lupus erythematosus (SLE)	Blood	Human sorted B cell
	GSM746730	Patient 5	Homo sapiens	Systemic lupus erythematosus (SLE)	Blood	Human sorted B cell
	GSM746731	Patient 6	Homo sapiens	Systemic lupus erythematosus (SLE)	Blood	Human sorted B cell
	GSM746732	Patient 7	Homo sapiens	Systemic lupus erythematosus (SLE)	Blood	Human sorted B cell
	GSM746733	Patient 8	Homo sapiens	Systemic lupus erythematosus (SLE)	Blood	Human sorted B cell
	GSM746734	Patient 9	Homo sapiens	Systemic lupus erythematosus (SLE)	Blood	Human sorted B cell
	GSM746735	Patient 10	Homo sapiens	Systemic lupus erythematosus (SLE)	Blood	Human sorted B cell
	GSM746736	Patient 11	Homo sapiens	Systemic lupus erythematosus (SLE)	Blood	Human sorted B cell
	GSM746737	Patient 12	Homo sapiens	Systemic lupus erythematosus (SLE)	Blood	Human sorted B cell
	GSM746738	Patient 13	Homo sapiens	Systemic lupus erythematosus (SLE)	Blood	Human sorted B cell
	GSM746739	Patient 14	Homo sapiens	Systemic lupus erythematosus (SLE)	Blood	Human sorted B cell
	GSM746740	Patient 15	Homo sapiens	Systemic lupus erythematosus (SLE)	Blood	Human sorted B cell
	GSM746741	Patient 16	Homo sapiens	Systemic lupus erythematosus (SLE)	Blood	Human sorted B cell
	GSM746742	Patient 17	Homo sapiens	Systemic lupus erythematosus (SLE)	Blood	Human sorted B cell
Control	GSM746743	Control 1	Homo sapiens	Control	Blood	Human sorted B cell
	GSM746744	Control 2	Homo sapiens	Control	Blood	Human sorted B cell
	GSM746745	Control 3	Homo sapiens	Control	Blood	Human sorted B cell
	GSM746746	Control 4	Homo sapiens	Control	Blood	Human sorted B cell
	GSM746747	Control 5	Homo sapiens	Control	Blood	Human sorted B cell
	GSM746748	Control 6	Homo sapiens	Control	Blood	Human sorted B cell
	GSM746750	Control 8	Homo sapiens	Control	Blood	Human sorted B cell
	GSM746751	Control 9	Homo sapiens	Control	Blood	Human sorted B cell
	GSM746752	Control 10	Homo sapiens	Control	Blood	Human sorted B cell

**TABLE 2:** Significantly upregulated and downregulated DEGs between two groups from GSE30153 dataset are tabulated.

GENE SYMBOL	log2FC	*p*-value
**Upregulating Genes**		
*EGR1*	1.22	0.00074
*DSE*	1.125	0.00291
*CD1C*	1.068	0.00053
*GPM6A*	1.052	0.00097
GPM6A*	1.043	0.002981
**Downregulating Genes**		
*RRM2*	–2.406	0.0027527
RRM2*	–2.152	0.0030096
*TYMS*	–1.923	0.0032032
*CD38*	–1.702	0.0031747
*CAV1*	–1.516	0.0048324
*MIR7110*	–1.4	0.0027212
*ELL2*	–1.354	0.0035256
*SLC44A1*	–1.219	0.0035298
*SAR1B*	–1.176	0.0049953
*MAN1A1*	–1.111	0.004425
*CHAC2*	–1.071	0.004354
*ERAP1*	–1.047	0.005321
*ARF4*	–1.044	0.0058274
*PDIA4*	–1.014	0.0041988

*The asterisk denotes the DEGs with two different probes from the dataset.

### Screening of Module and Construction of Interlinking PPI Network

**FIGURE 2:**
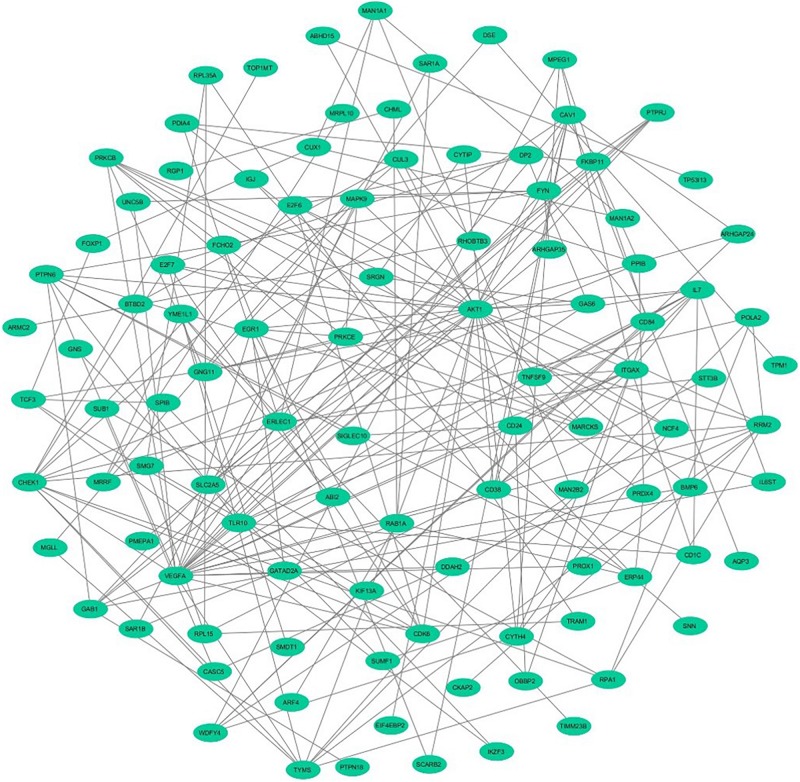
The network demonstrates the protein–protein interaction between the DEGs identified from GSE30153 using Cytoscape. The nodes represented as ellipse (robin’s blue) and edges as lines (gray).

**FIGURE 3:**
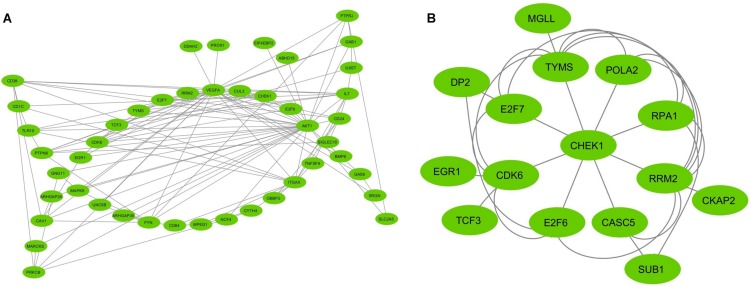
The MCODE (Molecular Complex Detection) plugin from Cytoscape analyzed the top two clusters derived from the network of interactions between protein and protein. **(A)** Cluster 1; **(B)** Cluster 2. The MCODE cluster score > 3. The nodes represented as ellipse (green) and edges as lines (gray).

To assess the protein–protein connections among the DEGs, we used the STRING tool to compute the protein interactions and plotted them using Cytoscape v3.7.2. Figure 2 depicts the PPI network with 103 nodes and 201 edges. The DEGs are represented as nodes, and the edges are interactions between the DEGs. A combined node score of >0.4 was considered to be significant. MCODE plugin v1.5.1 from Cytoscape was utilized to identify the densely interlinked regions within the protein network. As a result, we obtained the top two significant clusters from the DEGs protein network with MCODE scores of 5.043 and 3.625. A graphical representation of these clusters is shown in Figures 3A,B. The subnetworks, scores, number of nodes and edges, and node IDs are tabulated in Table 3.

**TABLE 3:** The interconnected regions are clustered from the GSE30153 dataset using MCODE plugin in Cytoscape.

Cluster	Score (density × No. of nodes)	Nodes	Edges	Node IDs
1	5.043	45	116	*OBBP2, IL6ST, CD1C, ABHD15, PTPRJ, ITGAX, UNC5B, TLR10, CD38, GAS6, NCF4, MAPK9, DDAH2, PTPN6, GAB1, ARHGAP24, CUL3, PROX1, CYTH4, E2F6, TNFSF9, VEGFA, TYMS, IL7, RRM2, PRKCB, MPEG1, MARCKS, SLC2A5, ARHGAP35, BMP6, TCF3, AKT1, EIF4EBP2, GNG11, CAV1, FYN, EGR1, SIGLEC10, CD24, CHEK1, E2F7, CD84, CDK6, SRGN*
2	3.625	15	29	*RRM2, CKAP2, MGLL, TCF3, SUB1, EGR1, POLA2, RPA1, CHEK1, E2F7, CASC5, DP2, E2F6, CDK6, TYMS*

### Enrichment Analysis by ClueGO

**FIGURE 4:**
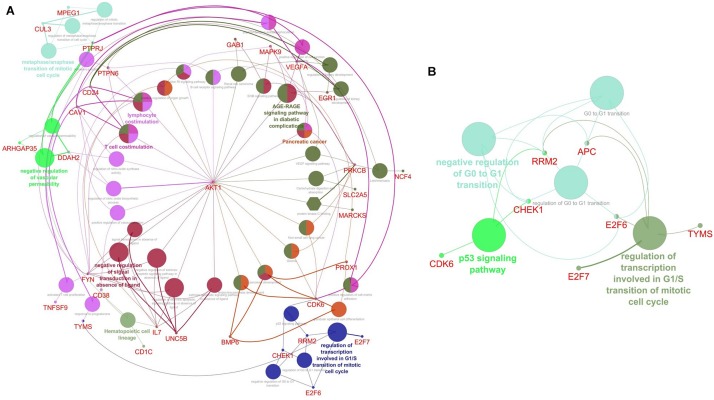
Visualization of Gene Ontology (GO) enrichment profiles from DEGs using Cytoscape software based on network analysis of ClueGO/CluePedia inferred from MCODE cluster 1 **(A)** and cluster 2 **(B)**. The plugin provides a combined enrichment analysis of clusters, including the GO biological process, molecular function, and pathway from KEGG. The GO term/pathway network connectivity defined by edges and functional clusters on genes shared between terms (kappa score ≥ 0.4) and displaying pathways only with *p*≤ 0.05. The size of the node indicates the *p*-value. The color code of nodes represents the functional group that they belong to. The most important functional terms specify the pathway names within each class are indicated in bold colored characters. **(A)** The network enrichment analysis of cluster 1. Each node constitutes a precise term for cluster 1; **(B)** The network enrichment analysis of cluster 2. Each node constitutes a precise term for cluster 2.

The top two subnetworks from MCODE were used as an input for analyzing the functional enrichment of PPI subnetworks using the ClueGO/CluePedia plugin from Cytoscape. In a biologically clustered subnetwork, ClueGO helps to visualize the biological terms of broad gene clusters. The subnetwork enrichment analyses of MCODE cluster 1 and cluster 2 are depicted in Figures 4A,B. For functional enrichment analysis, we set the statistical options based on a two-sided hypergeometric test with a Benjamini–Hochberg correction, *p* ≤ 0.05, and kappa scores ≥ 0.4 as criteria. The DEGs from cluster 1 were shown to be enriched mostly in T-cell costimulation (GO: 0031295), lymphocyte costimulation (GO: 0031294), negative regulation of vascular permeability (GO: 0043116), the metaphase/anaphase transition of the mitotic cell cycle (GO: 0007091), regulation of the transcription involved in the G1/S transition of the mitotic cell cycle (GO: 0000083), negative regulation of signal transduction in the absence of ligand (GO: 1901099), and KEGG pathways such as hematopoietic cell lineage (KEGG: 04640), B-cell receptor signaling pathway (KEGG: 04662), ErbB signaling pathway (KEGG: 04012), and AGE-RAGE (advanced glycation end products and receptor for AGE) signaling pathway in diabetic complications (Figure 4A). The DEGs from cluster 2 were mainly enriched in the regulation of the transcription involved in the G1/S transition of the mitotic cell cycle (GO: 0000083), the negative regulation of the G0 and G1 transitions (GO: 0070317), and the p53 signaling pathway (KEGG: 04115) (Figure 4B). The pathways that were activated in the enrichment analysis were highly related to B-cell pathophysiology, resulting in events associated with the immune system, vasculopathy, and kidney.

### Metacore^TM^ GeneGo for Enrichment Analysis of DEGs

**FIGURE 5:**
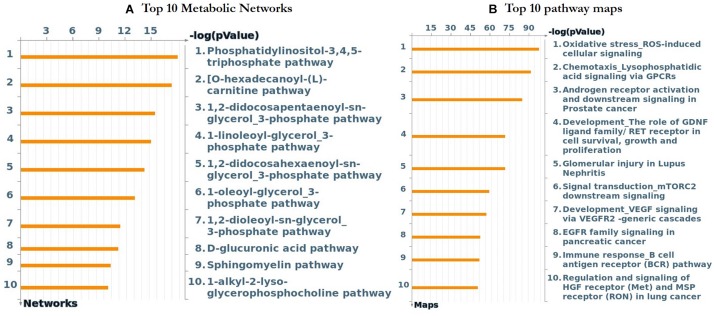
The top 10 metabolic networks and pathway maps were annotated using GeneGo enrichment analysis for the genes that are differentially expressed from SLE patients vs. healthy controls, respectively. **(A)** The content of these metabolic networks was annotated and defined by GeneGo Cortellis Solution software. Each process represents a pre-set network of protein interactions characteristic for the process, and sorting was performed for the metabolic networks that are statistically significant. **(B)** The pathway maps (canonical) of GeneGo display a series of signals and metabolic charts that cover human in a structured manner. The significant expression of a gene/protein represented in histogram height.

**FIGURE 6:**
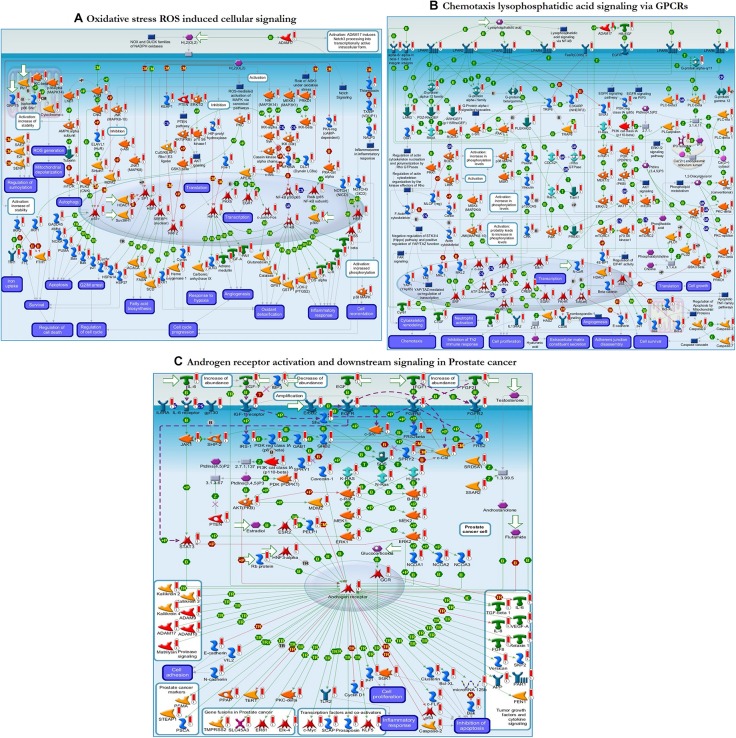
The enrichment analysis from GeneGo showed three regulated pathways with the highest score that are triggered in the SLE human sorted B-cells. **(A)** Oxidative stress ROS induced cellular signaling. **(B)** Chemotaxis lysophosphatidic acid signaling via G protein-coupled receptors (GPCRs). **(C)** Androgen receptor activation and downstream signaling in prostate cancer. The image depicts the protein and protein complexes that are well characterized as a specific symbol; laboratory data from all reports are correlated and shown on the maps as thermometer-like indicators. The red or blue color upward/downward thermometers indicate gene transcripts with upregulation/downregulation, respectively. The proteins connected by arrows demonstrate the stimulating and inhibitory effect of the protein. Further details are given at https://portal.genego.com/help/MC_legend.pdf.

Further functional enrichment analysis was carried out using Metacore^TM^ GeneGo software from Clarivate Analytics to comprehensively dissect the pathways associated with the DEGs. Using the functional ontology feature in GeneGo, the IDs of potential genes that were involved in the target pathways were identified. Based on hypergeometric *p*-values, the probability that the intersection of a gene set and associated ontological objects was random was evaluated. A decreased *p*-value indicated that the entity would be more significant to the DEGs, suggesting a better score. The functional enrichment analysis of the DEGs defined the top 10 metabolic networks, and canonical pathway maps are depicted in Figures 5A,B. For each classification, the significant statistical data rely on a low *p*-value. The pathway maps with the lowest *p*-value are shown in Figures 6A–C. These are the top-scoring signaling pathways based on the gene enrichment distribution, which emphasizes that the DEGs from human sorted B-cells are triggered via oxidative stress and ROS-induced cellular signaling (Figure 6A), chemotaxis and lysophosphatidic acid signaling via GPCRs (Figure 6B), and androgen receptor activation and downstream signaling in prostate cancer (Figure 6C). The well-distinguished proteins and complexes of proteins are shown as specific symbols^6^; all experimental data are displayed and have corresponding thermometer-like symbols on all the maps. The upregulated genes are indicated by a red thermometer facing upwards.

### Pathway Map Interaction Results From Clarivate

From the Metacore^TM^ results, we extracted the key genes from the enriched pathways that were differentially expressed, such as *EGR1*, *CD38*, *CAV1*, and *AKT1*. The differential expression of these genes was involved in the activation or inhibition of specific protein complexes in the enriched pathway maps (Figure 6 and Table 4). Early growth response 1 (EGR1) is a transcription factor that interacts with the *IGF-2*, *APEX*, *SRD5A1*, *CD44*, and *EGFR* genes and activates them through transcriptional regulation. The cyclic adenosine diphosphate (ADP) ribose hydrolase CD38 is an enzyme involved in the activation of the genes *Semaphorin 4D, CD19*, and *c-Cbl* through physical interactions. Caveolin 1 (CAV1) is a binding protein shown to interact with the *ErbB2, MDR1, HTR2A*, and *androgen receptor* genes, and inhibition or activation is followed by specific binding to its corresponding proteins. RAC-alpha serine/threonine-protein kinase (AKT1) is a protein kinase that interacts with the *FKHR, mTOR, Bcl-10, FOXO3A, HNF3-beta*, and *GSK3 beta* genes via phosphorylation, resulting in inhibition or activation. These genes were differentially expressed between sorted B-cells from controls and sorted B-cells from SLE patients and result in transcriptional regulation and inhibition of genes/proteins within the top-scored pathway maps.

**TABLE 4:** The interaction reports of key genes from pathway maps by Clarivate Analytics.

Network object “from”	Object type	Network object “to”	Object type	Effect	Mechanism	Link info	Input IDs	Signal	*P*-value	PMID
*EGR1*	Transcription factor	*IGF-2, APEX, SRD5A1, CD44, EGFR*	Receptor ligand, generic enzyme, generic enzyme, generic receptor, receptor with enzyme activity	Activation	Transcription regulation	EGR1 increases IGF II expression, EGR1 binds to gene APEX promoter and activates APEX expression, Egr-1 trans-activates the 5alpha-R1 promoter via the Egr-1-binding site at position −60/−54, Putative EGR1 binding site is found in gene CD44 promoter, EGR1 binds to gene EGFR promoter and activates EGFR expression.	*EGR1*	1	0.0032807	8584025; 9925986; 10606246; 11336542; 16043101; 19276347; 29092905; 29170465; 15788231; 15936112; 17194527; 18215136; 8628295; 9300687; 12670907; 15155664; 15923644; 19195913; 20357818; 25673149; 1417865; 11830539; 16750517; 17230532; 19032775; 20190820; 23763269
*CD38*	Generic enzyme	*SEMA4D, CD19, c-Cbl*	Generic receptor, generic binding protein, generic enzyme	Activation	Unspecified, Binding	CD31-induced activation of CD38 up-regulates Semaphorin 4D cell-surface expression in B cells, CD19/CD81 complex interacts with CD38 but this interaction is not required to induce proliferation in mouse B-lymphocytes, Fluorescence resource energy transfer and coimmunoprecipitation showed that c-Cbl and CD38 bind each other.	*CD38*	1	0.0031747	15613544; 17327405; 20570673; 22564057; 8695807; 18974118; 19635790
*CAV1*	Generic binding protein	*ErbB2, MDR1, HTR2A, Androgen receptor*	Receptor with enzyme activity, transporter, GPCR, transcription factor	Unspecified, Inhibition, activation	Binding	HER2 physically interacts with caveolin-1, Caveolin-1 interacts with p-gp, Down-regulation of caveolin-1 by siRNA reduced the interaction between p-gp and caveolin-1, followed by a decrease in [3H]-Taxol and [3H]-Vinblastine accumulation in RBE4 cells, Caveolin-1 physically interacts with HTR2A and increases its activity, Highly conserved 9 amino acid motif in the ligand binding domains (E domains) was identified in human/mouse ER alpha and ER beta, progesterone receptors A and B, and the androgen receptor. The localization sequence mediated palmitoylation of each SR, which facilitated caveolin-1 association, subsequent membrane localization, and steroid signaling.	*CAV1*	1	0.0048324	9374534; 9685399; 11697880; 22389470; 14622130; 15239129; 15498565; 17326770; 18485890; 19099191; 22389470; 25788263; 15190056; 8703009; 11278309; 17535799; 17940184; 18786521; 19931639; 22771325; 24375805
AKT1, AKT (PKB)	Protein kinase	*FKHR, mTOR, Bcl-10, FOXO3A, HNF3-beta, GSK3 beta*	Transcription factor, protein kinase, generic binding protein	Inhibition, activation	Phosphorylation	AKT1 phosphorylates FKHR1 and decreases its activity, Increased AKT phosphorylation regulates different metabolic pathways in liver, including increases in protein synthesis through activation of mTOR/p70 (S6kinase), AKT1 phosphorylates Bcl-10 and increases its activity, AKT1 phosphorylates FOXO3A and decreases its activity, AKT1 phosphorylates HNF3-beta and decreases its activity, AKT (PKB) inhibits GSK3 alpha by phosphorylation at Ser-9.	*AKT1*	1	0.0010146	10102273; 10358014; 10358075; 10377430; 11030146; 12393870; 16076959; 16099987; 16230533; 16603397; 17186497; 18388859; 18391970; 18420577; 18687691; 18786403; 19703413; 20940043; 21106439; 21157483; 21238503; 21407213; 21440577; 21708191; 21779512; 26053093; 27966458; 30413788; 10567225; 10910062; 11357143; 11438723; 12767043; 14970221; 15208671; 15549092; 16818631; 17660512; 18505677; 18566586; 18566587; 18678273; 21097843; 21177249; 21302298; 21343617; 22084251; 22595285; 23686889; 23872070; 26958938; 29221131; 16280327; 10102273; 12130673; 12767043; 17570479; 17577629; 17957242; 17960591; 18391970; 18687691; 19703413; 20223831; 20399660; 21106439; 21157483; 21440577; 21621563; 21708191; 21775285; 21779512; 24518891; 27966458; 14500912; 11584303; 11701324; 12124352; 12750378; 12808085; 14966899; 14985354; 15016802; 15297258

## Discussion

DNA microarrays and next generation sequencing (NGS) approaches are high-throughput technologies that have resulted in the emergence of new biomedical discoveries. Data from microarray and gene expression profiles have enabled a deeper understanding of the intrinsic molecular pathways of complex mechanisms of biological systems and their responses (Russo et al., 2003; Babu, 2004; Perez-Diez et al., 2013; Kumar et al., 2019). It is therefore highly relevant to examine the peripheral B-cell transcriptomes of SLE patients and healthy controls to determine genes that are differentially regulated and their target pathways. Our current study extracted DEGs from 17 SLE patients and 9 healthy controls from the GEO database (GSE30153) (Garaud et al., 2011). The top 250 DEGs were identified, including 4 upregulated and 13 downregulated genes from the groups through bioinformatics strategies (Table 2 and Supplementary Table S1). These identified DEGs were subjected to ClueGO and GeneGo Metacore^TM^ analysis for GO and pathway annotation, and constructed the interacting networks of PPI and used for cluster analysis. In the network, the nodes were considered proteins, and the edges were their interactions. Using network topology features, the PPI network can be analyzed to distinguish the core proteins that are involved in the pathways (Barabási and Oltvai, 2004; Ideker and Sharan, 2008; Keskin et al., 2016; Kumar et al., 2019). The identified DEGs from the present study were analyzed with STRING to exploit the complex interactions between the DEGs via text mining, evidence from experiments, and repositories (Figure 2). We performed module screening of the PPI networks using the MCODE plugin from Cytoscape. As a result, we obtained significant clusters that are densely interlinked regions in the PPI network (Figures 3A,B). Screening of these clusters from the network might help to identify the essential genes that are involved in the pathogenesis and progression of SLE. The obtained clusters mostly contained protein complexes or proteins present in the pathways in the PPI network, and cluster visualization is essential for comprehending the properties of the network functionally and systematically (Krogan et al., 2006; Rahman et al., 2013).

Furthermore, to identify the functional enrichment of these subnetworks from MCODE, we implemented the ClueGO plugin for analysis. This revealed that the DEGs were enriched in most essential pathways, which are highly associated with the immune system. The GO and KEGG enrichment analyses of the DEGs from cluster 1 showed that they were mostly enriched in T-cell costimulation, lymphocyte costimulation, negative regulation of vascular permeability, the metaphase/anaphase transition of the mitotic cell cycle, regulation of the transcription involved in the G1/S transition of mitotic cell cycle, the hematopoietic cell lineage, the B-cell receptor signaling pathway, the ErbB signaling pathway, the AGE-RAGE signaling pathway in diabetic complications, and pancreatic cancer. Interestingly, the costimulation of T-cell and lymphocyte receptors is recognized to be important in SLE pathogenesis by enabling communicating with B-cells for the production of autoantibodies (Shlomchik et al., 2001; Mak and Kow, 2014). In SLE, negative regulation of vascular permeability may be induced by different mechanisms; the dysregulated genes from the cluster 1 subnetwork might lead to endothelial cell damage and vasculopathy (Favero et al., 2014; Lee et al., 2019). The differential cell signaling results in the recruitment of various proteins and inappropriate activation of B-cells (Zhou et al., 2009; Comte et al., 2015). Oxidative stress is common in inflammatory disorders and results in the increased production of reactive carbonyl groups that are partially converted to AGEs, and the DEGs in the AGE-RAGE signaling pathway might also be involved in the accumulation of AGEs in SLE patients and lead to diabetic complications (de Leeuw et al., 2007; Li et al., 2007; Kurien and Scofield, 2008; Nienhuis et al., 2008). Interestingly, our enrichment analysis found that the identified differential expression of the genes (*AKT1*, *VEGFA*, *CDK6*, and *MAPK9*) that were involved in the risk of developing pancreatic cancer in SLE patients was due to chronic inflammation, suggesting that these genes might be involved in the pathogenesis of SLE. Our findings are therefore consistent with the roles of genes that are differentially expressed in SLE-causing pathways (Figure 4A). The enrichment analysis of the cluster 2 subnetwork showed that the DEGs were mostly enriched in the regulation of transcription involved in the G1/S transition of the mitotic cell cycle, the negative regulation of the G0 and G1 transitions, and the p53 signaling pathway. It has been reported that the proliferation of T-cells is followed by lowered levels of cyclin-dependent kinase (CDK) inhibitors, and alterations in the expression of CDKs in the G0/G1 phase were seen in the lymphocytes of SLE patients (Yamauchi and Bloom, 1997; Tang et al., 2009). The DEGs involved in the cluster 2 subnetwork might negatively regulate these pathways. Alterations in cyclin-CDK complex behavior and cyclin-dependent kinase inhibitors (CDKIs) have been reported to alter the proliferation of T-cells, oxidative stress, and immune responses (Santiago-Raber et al., 2001; Tang et al., 2009). p53 signaling is essential for various cellular mechanisms, and defects in this signaling pathway are associated with SLE development. Considerably elevated levels of p53 protein are found in SLE patients with active inflammatory disorders (Miret et al., 2003; Veeranki and Choubey, 2010). Apoptosis dysregulation appears to be another cause of SLE pathogenesis because the possible sources of autoantigens are cell debris from apoptosis in SLE, and excessive cellular senescence of the immune cells, especially T-cells, was reported in SLE patients with peripheral blood mononuclear cells (PBMCs) and skin lesions (Colonna et al., 2014; Sáenz-Corral et al., 2015). Thus, our identified DEGs (*RRM2*, *APC*, *CHEK1*, *E2F6*, *TYMS*, *E2F7*, and *CDK6*) from the cluster 2 subnetwork are highly related to and consistent with the members of the signaling pathways associated with the immune system, apoptosis, the cell cycle, and vasculopathy.

To clearly define the interactions between the proteins and signaling pathways examined from the interpretation of STRING, Cytoscape, MCODE, and ClueGO analyses, we implemented the GeneGo Metacore software, which incorporates extensive data on metabolic signaling pathways and their regulatory mechanisms and contains accurately complied networks of biological systems. By utilizing the GeneGo Metacore software, we obtained a detailed description of the DEGs that participate in SLE pathogenesis based on the determined *p*-values. Among the top 10 metabolic networks, the phosphatidylinositol-3,4,5-triphosphate pathway, O-hexadecanoyl-(L)-carnitine pathway, 1,2-didocosapentaenoyl-sn-glycerol 3-phosphate pathway, and 1-linoleoyl-glycerol-3-phosphate pathway were profoundly enriched and significant in the SLE DEGs (Figure 5A). The increased activity of phosphatidylinositol-3,4,5-triphosphate stimulates essential cell signaling pathways such as the pathways involved in cell division, survival, and the rapid increase in T-lymphocytes in SLE (Comte et al., 2015). PI3K (phosphatidylinositol 3-kinase) is a protein kinase that phosphorylates phosphatidylinositol 4,5-phosphate to regulate the signaling of T-lymphocytes; an increased amount of PI3K was also observed in an animal model of lupus (Liu et al., 1998; Grolleau et al., 2000; Niculescu et al., 2003; Joseph et al., 2014). Modification of the carnitine signaling pathway results in various organ failures by producing effective responses to pathogens (Famularo and De Simone, 1995; Famularo et al., 2004). Thus, the DEGs involved in the O-hexadecanoyl-(L)-carnitine pathway might lead to increased immune responses. In addition, the top three pathways associated with the DEGs of sorted B-cells from SLE patients were mostly enriched in oxidative stress- and ROS-induced cellular signaling (Figure 6A), chemotaxis and lysophosphatidic acid signaling via GPCRs (Figure 6B), and androgen receptor activation and downstream signaling in prostate cancer (Figure 6C). Recent findings have shown that oxidative stress and ROS induce molecular alterations that have adverse effects in SLE patients (Choi et al., 2016; Tsokos et al., 2016; Lightfoot et al., 2017). Elevated oxidative stress in SLE patients leads to the accumulation of higher amounts of oxidative lipoproteins, which are harmful in zebrafish models and cause additional oxidative damage to the system (Chung et al., 2007; Park et al., 2016; Lightfoot et al., 2017). Interestingly, our study identified the *EGR1* gene as downregulated in the SLE patients in comparison to controls, and it also plays a role in ROS signaling. This clearly indicates that *EGR1* might be required to maintain the oxidative stress and ROS signaling pathways.

Moreover, the DEGs involved in the oxidative stress signaling pathway might contribute to peripheral neuropathy, damage to blood vessels, and cardiovascular events, which are the prominent clinical conditions found in SLE patients. Chemotaxis and lysophosphatidic acid (LPA) signaling are essential pathways in autoimmune inflammatory disorders, and GPCRs are responsible for regulating immune cells via LPA receptors (Yang et al., 2005; Skoura and Hla, 2009). *G2A* gene knockout resulted in the hyperresponsiveness of T-cells to T-cell receptor stimulation, manifesting as an increased proliferation of T-cells, which may promote inflammatory phenotypes in G2A-deficient mice (Le et al., 2001). Various studies have suggested that LPA plays a vital role in atherosclerosis progression and development by promoting neutrophil and monocyte adherence and enhancing inflammatory events (Siess et al., 1999; Smyth et al., 2008; Skoura and Hla, 2009). The androgen receptor (AR) is a transcription factor that is activated by a ligand and is essential for cells targeted by the androgen response (Robeva et al., 2013; Gubbels Bupp and Jorgensen, 2018). AR also regulates immune function in SLE via transcriptional regulation of various genes. Our study identified the transcription factor *AR*, which positively regulates the *c-Myc, SCAP, prosaposin*, and *KLF5* genes, which are responsible for inflammatory responses, and promotes tumor growth factors and cytokine signaling when activated (Figure 6C). The enriched terms from ClueGO modules and the GeneGo-identified terms correlated well in this study and validate the significance of the findings from the pathway maps. The combined results from these two enrichment analyses suggest that B-cells from SLE patients and B-cells from healthy controls undergo differential gene expression associated with positive regulation of kidney development, the hematopoietic cell lineage, positive regulation of vasoconstriction, T-cell costimulation, and regulation of the transcription involved in the G1/S transition of the mitotic cell cycle.

Furthermore, the interaction results from the GeneGo analysis provided the essential genes (*EGR1*, *CD38*, *CAV1*, and *AKT1*) from the pathway maps constructed from the DEGs. Among them, *EGR1* (early growth response 1) is a transcription factor shown to interact with the *IGF-2* (insulin-like growth factor 2), *APEX* (apurinic/apyrimidinic endodeoxyribonuclease 1), *SRD5A1* (steroid 5 alpha-reductase 1), *CD44* (cell surface glycoprotein CD44), and *EGFR* (epidermal growth factor receptor) genes and transcriptionally regulate them by activating or promoting their expression in sorted B-cells from patients with SLE (Liu et al., 1995; Recio and Merlino, 2003; Lee et al., 2005; Pines et al., 2005; Blanchard et al., 2007; Rui et al., 2008; Cullen et al., 2010; Sauer et al., 2010). The cyclic ADP ribose hydrolase (CD38) is also known as cluster of differentiation 38 protein, can be found on several immune cells, and activates SEMA4D (semaphorin-4D or cluster of differentiation 100), CD19 (B-lymphocyte antigen CD19 or cluster of differentiation 19), and c-Cbl (Casitas B-lineage lymphoma proto-oncogene) (Deaglio et al., 2005, 2007; Shen and Yen, 2008; Vences-Catalán et al., 2012). These interactions with *CD38* result in the activation of B-lymphocytes and increase immune responses in SLE patients. The protein caveolin 1 (CAV1) has been shown to interact with the ErbB2 (Erb-B2 receptor tyrosine kinase 2), MDR1 (multidrug resistance protein 1), HTR2A (5-hydroxytryptamine receptor 2A), and AR (androgen receptor) genes. Several studies have suggested that caveolin 1 physically interacts with HER2, p-gp, HTR2A, and AR and activates/inhibits them by binding to their specific caveolin-binding motif (Couet et al., 1997; Lu et al., 2001; Razani and Lisanti, 2001; Bhatnagar et al., 2004; Bennett et al., 2010, 2014; Yu et al., 2012). AKT1 is a protein kinase that interacts with *FKHR* (Forkhead box protein O1), mTOR (mechanistic target of rapamycin), Bcl-10 (B-cell lymphoma/leukemia 10), FOXO3A (Forkhead box O3), HNF3-beta (hepatocyte nuclear factor 3-beta), and GSK3 beta (glycogen synthase kinase three beta). The protein kinase AKT1 inhibits FKHR via phosphorylation and decreases its activity (Biggs et al., 1999; Rena et al., 1999; Tang et al., 1999; Hay, 2011), whereas it increases its activity via phosphorylation of mTOR (Navé et al., 1999; Sekuliæ et al., 2000; Ikenoue et al., 2008; Thirumal Kumar et al., 2019). Additionally, AKT1 phosphorylates Bcl-10 at the specific residues Ser231 and Ser218, increasing its activity (Yeh et al., 2006), while it inhibits the action of FOXO3A via phosphorylation and decreases its activity, increasing the survival of cells (Brunet et al., 1999; Linding et al., 2007; Calnan and Brunet, 2008; Li et al., 2008; Tzivion et al., 2011). AKT1 decreases HNF3-beta activity by phosphorylating it at Thr156 (Wolfrum et al., 2003), whereas phosphorylation of GSK3-beta by AKT1 occurs at Ser9 to inhibit its activity (Brazil and Hemmings, 2001; Salas et al., 2004; Kuemmerle, 2005; Shin et al., 2006; Markou et al., 2008). This suggests the vital genes we identified from the DEGs of patients with SLE play essential roles in the development and progression of SLE via different signaling pathways to increase autoimmune responses.

**FIGURE 7:**
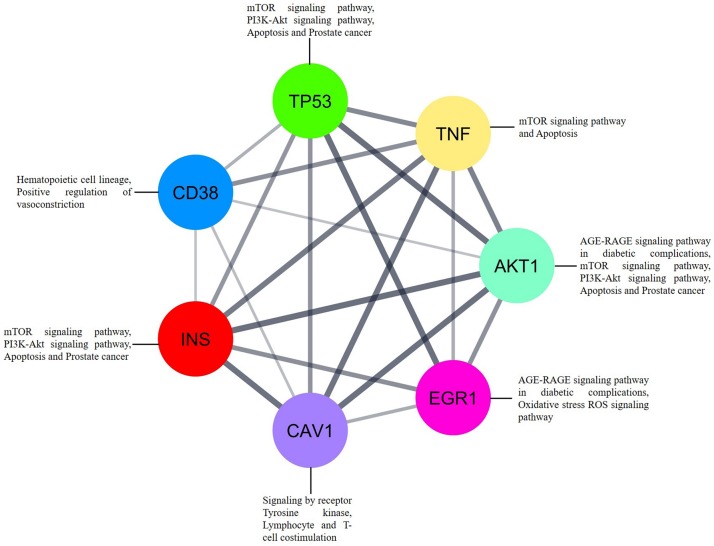
The interrelation analysis of genes *EGR1*, *CD38*, *CAV1*, and *AKT1* that strongly associated to SLE. Each gene involved in different pathways via interacting to each other. Inbuilt color code was provided to all the genes based on the STRING tool from Cytoscape.

In addition to the interaction analysis, we carried out interrelation analysis for the essential genes to determine the relationships between the genes, which implicitly or explicitly interacted with each other. Interestingly, the identified genes indirectly communicated with each other via molecular signaling pathways related to mTOR signaling, apoptosis, PI3K-Akt signaling, the hematopoietic cell lineage, positive regulation of vasoconstriction, signaling by receptor tyrosine kinases, AGE-RAGE signaling, and lymphocyte and T-cell costimulation (Figure 7). *EGR1* and *AKT1* are directly involved in oxidative stress via ROS and AGE-RAGE signaling, whereas *CAV1* is directly involved in tyrosine kinase receptor signaling and lymphocyte and T-cell costimulation. *CD38* is directly associated with the hematopoietic cell lineage and positive regulation of vasoconstriction. Overall, the dysregulation of the indicated pathways in SLE patients is a result of differential gene expression. The essential genes are differentially expressed between cells from patients with SLE and cells from healthy controls and are present in important signaling cascades, which could be a crucial factor for SLE development.

## Conclusion

Taken together, the results of our comprehensive bioinformatics analysis showed that the DEGs identified between sorted B-cells from patients with SLE sorted B-cells from controls could play a significant role in the growth, progression, and development of SLE. This study identified 4 upregulated and 13 downregulated genes, including essential genes (*EGR1*, *CD38*, *CAV1*, and *AKT1*), from the pathway enrichment analysis. Indeed, the identified pathways from the enrichment analysis were strongly related to the immune system, vasculopathy, cardiovascular functions, and inflammatory responses, which are processes that can lead to the development of SLE. The broad understanding of SLE pathophysiology from this study will allow us to identify and develop therapies targeting SLE and contribute to personalized treatment strategies. Collectively, the study findings could aid in enhancing our understanding of the fundamental molecular processes of SLE and provide possible strategies for early diagnosis in SLE; in addition, combinatorial therapeutic strategies using oxidative stress and ROS cellular signaling and lysophosphatidic acid signaling via GPCRs might have symbiotic effects on the molecular events in SLE.

## Data Availability Statement

Publicly available datasets were analyzed in this study. This data can be found here: https://www.ncbi.nlm.nih.gov/geo/query/acc.cgi?acc=GSE30153.

## Author Contributions

SU, DT, HZ, and CG were involved in the design of the study and the acquisition, analysis, and interpretation of the data. SU, DT, CG, SY, NY, MS, and HZ were involved in the interpretation of the data and drafting the manuscript. CG, RS, and HZ supervised the entire study and were involved in study design, the acquisition, analysis, and interpretation of the data, and drafting the manuscript. The manuscript was reviewed and approved by all the authors.

## Conflict of Interest

The authors declare that the research was conducted in the absence of any commercial or financial relationships that could be construed as a potential conflict of interest.

## Footnotes

1 https://www.ncbi.nlm.nih.gov/geo/

2 https://paolo.shinyapps.io/ShinyVolcanoPlot/

3 http://www.string-db.org/

4 http://www.cytoscape.org/

5 https://clarivate.com/products/metacore/

6 https://portal.genego.com/help/MC_legend.pdf

## References

[B1] Alibés A. Yankilevich P. Cañada A. Díaz-Uriarte R. (2007). IDconverter and IDClight: conversion and annotation of gene and protein IDs. *BMC Bioinformatics* 8:9. 10.1186/1471-2105-8-9 17214880 PMC1779800

[B2] Aubert J. Bar-Hen A. Daudin J. J. Robin S. (2004). Determination of the differentially expressed genes in microarray experiments using local FDR. *BMC Bioinformatics* 5:125. 10.1186/1471-2105-5-125 15350197 PMC520755

[B3] Babu M. M. (2004). *An Introduction to Microarray Data Analysis.* Cambridge: Horizon Bioscience, 225–249.

[B4] Bader G. D. Hogue C. W. (2003). An automated method for finding molecular complexes in large protein interaction networks. *BMC Bioinformatics* 4:2. 10.1186/1471-2105-4-2 12525261 PMC149346

[B5] Barabási A.-L. Oltvai Z. N. (2004). Network biology: understanding the cell’s functional organization. *Nat. Rev. Genet.* 5 101–113. 10.1038/nrg1272 14735121

[B6] Barrett T. Wilhite S. E. Ledoux P. Evangelista C. Kim I. F. Tomashevsky M. (2013). NCBI GEO: archive for functional genomics data sets–update. *Nucleic Acids Res.* 41 D991–D995. 10.1093/nar/gks1193 23193258 PMC3531084

[B7] Benjamini Y. Hochberg Y. (1995). Controlling the false discovery rate: a practical and powerful approach to multiple testing. *J. R. Statist. Soc. Ser. B (Methodol.)* 57 289–300.

[B8] Bennett N. C. Gardiner R. A. Hooper J. D. Johnson D. W. Gobe G. C. (2010). Molecular cell biology of androgen receptor signalling. *Int. J. Biochem. Cell Biol.* 42 813–827. 10.1016/j.biocel.2009.11.013 19931639

[B9] Bennett N. C. Hooper J. D. Johnson D. W. Gobe G. C. (2014). Expression profiles and functional associations of endogenous androgen receptor and caveolin-1 in prostate cancer cell lines. *Prostate* 74 478–487. 10.1002/pros.22767 24375805

[B10] Bentham J. Morris D. L. Cunninghame Graham D. S. Pinder C. L. Tombleson P. Behrens T. W. (2015). Genetic association analyses implicate aberrant regulation of innate and adaptive immunity genes in the pathogenesis of systemic lupus erythematosus. *Nat. Genet.* 47 1457–1464. 10.1038/ng.3434 26502338 PMC4668589

[B11] Bhatnagar A. Sheffler D. J. Kroeze W. K. Compton-Toth B. Roth B. L. (2004). Caveolin-1 interacts with 5-HT2A serotonin receptors and profoundly modulates the signaling of selected Gαq-coupled protein receptors. *J. Biol. Chem.* 279 34614–34623. 10.1074/jbc.M404673200 15190056

[B12] Biggs W. H. Meisenhelder J. Hunter T. Cavenee W. K. Arden K. C. (1999). Protein kinase B/Akt-mediated phosphorylation promotes nuclear exclusion of the winged helix transcription factor FKHR1. *Proc. Natl. Acad. Sci. U.S.A.* 96 7421–7426. 10.1073/pnas.96.13.7421 10377430 PMC22101

[B13] Bindea G. Mlecnik B. Hackl H. Charoentong P. Tosolini M. Kirilovsky A. (2009). ClueGO: a cytoscape plug-in to decipher functionally grouped gene ontology and pathway annotation networks. *Bioinformatics* 25 1091–1093. 10.1093/bioinformatics/btp101 19237447 PMC2666812

[B14] Blanchard Y. Seenundun S. Robaire B. (2007). The promoter of the rat 5α-reductase type 1 gene is bidirectional and Sp1-dependent. *Mol. Cell. Endocrinol.* 264 171–183. 10.1016/j.mce.2006.11.007 17194527

[B15] Borrebaeck C. A. K. Sturfelt G. Wingren C. (2014). “Recombinant antibody microarray for profiling the serum proteome of SLE,” in *Systemic Lupus Erythematosus: Methods and Protocols* Methods in Molecular Biology, eds Eggleton P. Ward F. J. (New York, NY: Springer), 67–78. 10.1007/978-1-4939-0326-9_6 24497355

[B16] Brazil D. P. Hemmings B. A. (2001). Ten years of protein kinase B signalling: a hard Akt to follow. *Trends Biochem. Sci.* 26 657–664. 10.1016/S0968-0004(01)01958-2 11701324

[B17] Brunet A. Bonni A. Zigmond M. J. Lin M. Z. Juo P. Hu L. S. (1999). Akt promotes cell survival by phosphorylating and inhibiting a forkhead transcription factor. *Cell* 96 857–868. 10.1016/S0092-8674(00)80595-4 10102273

[B18] Calnan D. R. Brunet A. (2008). The FoxO code. *Oncogene* 27 2276–2288. 10.1038/onc.2008.21 18391970

[B19] Choi S.-C. Titov A. A. Sivakumar R. Li W. Morel L. (2016). Immune cell metabolism in systemic lupus erythematosus. *Curr. Rheumatol. Rep.* 18:66. 10.1007/s11926-016-0615-7 27709413

[B20] Chung C. P. Avalos I. Oeser A. Gebretsadik T. Shintani A. Raggi P. (2007). High prevalence of the metabolic syndrome in patients with systemic lupus erythematosus: association with disease characteristics and cardiovascular risk factors. *Ann. Rheum. Dis.* 66 208–214. 10.1136/ard.2006.054973 16901956 PMC1798504

[B21] Colonna L. Lood C. Elkon K. (2014). Beyond apoptosis in lupus. *Curr Opin Rheumatol.* 26 459–466. 10.1097/BOR.0000000000000083 25036095 PMC4272326

[B22] Comte D. Karampetsou M. P. Tsokos G. C. (2015). T cells as a therapeutic target in SLE. *Lupus* 24 351–363. 10.1177/0961203314556139 25801878 PMC4372812

[B23] Costa-Reis P. Sullivan K. E. (2013). Genetics and epigenetics of systemic lupus erythematosus. *Curr. Rheumatol. Rep.* 15:369. 10.1007/s11926-013-0369-4 23943494

[B24] Couet J. Sargiacomo M. Lisanti M. P. (1997). Interaction of a receptor tyrosine kinase, EGF-R, with caveolins. caveolin binding negatively regulates tyrosine and serine/threonine kinase activities. *J. Biol. Chem.* 272 30429–30438. 10.1074/jbc.272.48.30429 9374534

[B25] Cui Y. Sheng Y. Zhang X. (2013). Genetic susceptibility to SLE: Recent progress from GWAS. *J. Autoimm.* 41 25–33. 10.1016/j.jaut.2013.01.008 23395425

[B26] Cullen E. M. Brazil J. C. O’Connor C. M. (2010). Mature human neutrophils constitutively express the transcription factor EGR-1. *Mol. Immunol.* 47 1701–1709. 10.1016/j.molimm.2010.03.003 20363028

[B27] Dang J. Li J. Xin Q. Shan S. Bian X. Yuan Q. (2016). Gene–gene interaction of ATG5, ATG7, BLK and BANK1 in systemic lupus erythematosus. *Int. J. Rheum. Dis.* 19 1284–1293. 10.1111/1756-185X.12768 26420661

[B28] de Leeuw K. Graaff R. de Vries R. Dullaart R. P. Smit A. J. Kallenberg C. G. (2007). Accumulation of advanced glycation endproducts in patients with systemic lupus erythematosus. *Rheumatology (Oxford)* 46 1551–1556. 10.1093/rheumatology/kem215 17848401

[B29] Deaglio S. Vaisitti T. Bergui L. Bonello L. Horenstein A. L. Tamagnone L. (2005). CD38 and CD100 lead a network of surface receptors relaying positive signals for B-CLL growth and survival. *Blood* 105 3042–3050. 10.1182/blood-2004-10-3873 15613544

[B30] Deaglio S. Vaisitti T. Billington R. Bergui L. Omede’ P. Genazzani A. A. (2007). CD38/CD19: a lipid raft–dependent signaling complex in human B cells. *Blood* 109 5390–5398. 10.1182/blood-2006-12-061812 17327405

[B31] Deng Y. Tsao B. P. (2010). Genetic susceptibility to systemic lupus erythematosus in the genomic era. *Nat. Rev. Rheumatol.* 6 683–692. 10.1038/nrrheum.2010.176 21060334 PMC3135416

[B32] Ducreux J. Houssiau F. A. Vandepapelière P. Jorgensen C. Lazaro E. Spertini F. (2016). Interferon α kinoid induces neutralizing anti-interferon α antibodies that decrease the expression of interferon-induced and B cell activation associated transcripts: analysis of extended follow-up data from the interferon α kinoid phase I/II study. *Rheumatology (Oxford)* 55 1901–1905. 10.1093/rheumatology/kew262 27354683 PMC5034220

[B33] Edgar R. Domrachev M. Lash A. E. (2002). Gene expression omnibus: NCBI gene expression and hybridization array data repository. *Nucleic Acids Res.* 30 207–210. 10.1093/nar/30.1.207 11752295 PMC99122

[B34] Famularo G. De Simone C. (1995). A new era for carnitine? *Immunol. Today* 16 211–213. 10.1016/0167-5699(95)80159-6 7779248

[B35] Famularo G. Simone C. de, Trinchieri V. Mosca L. (2004). Carnitines and its congeners: a metabolic pathway to the regulation of immune response and inflammation. *Ann. N. Y. Acad. Sci.* 1033 132–138. 10.1196/annals.1320.012 15591010

[B36] Favero G. Paganelli C. Buffoli B. Rodella L. F. Rezzani R. (2014). Endothelium and its alterations in cardiovascular diseases: life style intervention. *Biomed. Res. Int.* 2014:801896. 10.1155/2014/801896 24719887 PMC3955677

[B37] Garaud J.-C. Schickel J.-N. Blaison G. Knapp A.-M. Dembele D. Ruer-Laventie J. (2011). B cell signature during inactive systemic lupus is heterogeneous: toward a biological dissection of lupus. *PLoS One* 6:e23900. 10.1371/journal.pone.0023900 21886837 PMC3160348

[B38] Grolleau A. Kaplan M. J. Hanash S. M. Beretta L. Richardson B. (2000). Impaired translational response and increased protein kinase PKR expression in T cells from lupus patients. *J. Clin. Invest.* 106 1561–1568. 10.1172/JCI9352 11120763 PMC381471

[B39] Gubbels Bupp M. R. Jorgensen T. N. (2018). Androgen-Induced Immunosuppression. *Front. Immunol.* 9:794. 10.3389/fimmu.2018.00794 29755457 PMC5932344

[B40] Gurevitz S. Snyder J. Wessel E. Frey J. Williamson B. (2013). Systemic lupus erythematosus: a review of the disease and treatment options. *Consult. Pharm.* 28 110–121. 10.4140/TCP.n.2013.110 23395811

[B41] Harley I. T. W. Kaufman K. M. Langefeld C. D. Harley J. B. Kelly J. A. (2009). Genetic susceptibility to SLE: new insights from fine mapping and genome-wide association studies. *Nat. Rev. Genet.* 10 285–290. 10.1038/nrg2571 19337289 PMC2737697

[B42] Hay N. (2011). Interplay between FOXO, TOR, and Akt. *Biochim. Biophys. Acta (BBA) Mol. Cell Res.* 1813 1965–1970. 10.1016/j.bbamcr.2011.03.013 21440577 PMC3427795

[B43] Ideker T. Sharan R. (2008). Protein networks in disease. *Genome Res.* 18 644–652. 10.1101/gr.071852.107 18381899 PMC3863981

[B44] Ikenoue T. Inoki K. Yang Q. Zhou X. Guan K.-L. (2008). Essential function of TORC2 in PKC and Akt turn motif phosphorylation, maturation and signalling. *EMBO J.* 27 1919–1931. 10.1038/emboj.2008.119 18566587 PMC2486275

[B45] Irizarry R. A. Bolstad B. M. Collin F. Cope L. M. Hobbs B. Speed T. P. (2003). Summaries of affymetrix genechip probe level data. *Nucleic Acids Res.* 31:e15. 10.1093/nar/gng015 12582260 PMC150247

[B46] Joseph N. Reicher B. Barda-Saad M. (2014). The calcium feedback loop and T cell activation: How cytoskeleton networks control intracellular calcium flux. *Biochim. Biophys. Acta (BBA) Biomemb.* 1838 557–568. 10.1016/j.bbamem.2013.07.009 23860253

[B47] Keskin O. Tuncbag N. Gursoy A. (2016). Predicting protein–protein interactions from the molecular to the proteome level. *Chem. Rev.* 116 4884–4909. 10.1021/acs.chemrev.5b00683 27074302

[B48] Krogan N. J. Cagney G. Yu H. Zhong G. Guo X. Ignatchenko A. (2006). Global landscape of protein complexes in the yeast Saccharomyces cerevisiae. *Nature* 440 637–643. 10.1038/nature04670 16554755

[B49] Kuemmerle J. F. (2005). Endogenous IGF-I protects human intestinal smooth muscle cells from apoptosis by regulation of GSK-3β activity. *Am. J. Physiol. Gastroin. Liver Physiol.* 288 G101–G110. 10.1152/ajpgi.00032.2004 15297258

[B50] Kumar S. U. Kumar D. T. Siva R. Doss C. G. P. Zayed H. (2019). Integrative bioinformatics approaches to map potential novel genes and pathways involved in ovarian cancer. *Front. Bioeng. Biotechnol.* 7:391. 10.3389/fbioe.2019.00391 31921802 PMC6927934

[B51] Kurien B. T. Scofield R. H. (2008). Autoimmunity and oxidatively modified autoantigens. *Autoimmun. Rev.* 7 567–573. 10.1016/j.autrev.2008.04.019 18625446 PMC2585037

[B52] Le L. Q. Kabarowski J. H. Weng Z. Satterthwaite A. B. Harvill E. T. Jensen E. R. (2001). Mice lacking the orphan G protein-coupled receptor G2A develop a late-onset autoimmune syndrome. *Immunity* 14 561–571. 10.1016/s1074-7613(01)00145-5 11371358

[B53] Lee W.-F. Wu C.-Y. Yang H.-Y. Lee W.-I. Chen L.-C. Ou L.-S. (2019). Biomarkers associating endothelial Dysregulation in pediatric-onset systemic lupus erythematous. *Pediat. Rheumatol.* 17:69. 10.1186/s12969-019-0369-7 31651352 PMC6814049

[B54] Lee Y.-S. Jang H.-S. Kim J.-M. Lee J.-S. Lee J.-Y. Kim K. L. (2005). Adenoviral-mediated delivery of early growth response factor-1 gene increases tissue perfusion in a murine model of hindlimb ischemia. *Mol. Ther.* 12 328–336. 10.1016/j.ymthe.2005.03.027 16043101

[B55] Li J.-T. Hou F.-F. Guo Z.-J. Shan Y.-X. Zhang X. Liu Z.-Q. (2007). Advanced glycation end products upregulate C-reactive protein synthesis by human hepatocytes through stimulation of monocyte IL-6 and IL-1β production. *Scand. J. Immunol.* 66 555–562. 10.1111/j.1365-3083.2007.02001.x 18027444

[B56] Li Y. Wang Z. Kong D. Li R. Sarkar S. H. Sarkar F. H. (2008). Regulation of Akt/FOXO3a/GSK-3β/AR signaling network by isoflavone in prostate cancer cells. *J. Biol. Chem.* 283 27707–27716. 10.1074/jbc.M802759200 18687691 PMC2562074

[B57] Lightfoot Y. L. Blanco L. P. Kaplan M. J. (2017). Metabolic abnormalities and oxidative stress in lupus. *Curr. Opin. Rheumatol.* 29 442–449. 10.1097/BOR.0000000000000413 28639951 PMC5586499

[B58] Linding R. Jensen L. J. Ostheimer G. J. Vugt M. A. T. M. van Jørgensen C. Miron I. M. (2007). Systematic discovery of in vivo phosphorylation networks. *Cell* 129 1415–1426. 10.1016/j.cell.2007.05.052 17570479 PMC2692296

[B59] Liu K.-Q. Bunnell S. C. Gurniak C. B. Berg L. J. (1998). T cell receptor–initiated calcium release is uncoupled from capacitative calcium entry in itk-deficient T cells. *J. Exp. Med.* 187 1721–1727. 10.1084/jem.187.10.1721 9584150 PMC2212298

[B60] Liu Z. Mittanck D. W. Kim S. Rotwein P. (1995). Control of insulin-like growth factor-II/mannose 6-phosphate receptor gene transcription by proximal promoter elements. *Mol. Endocrinol.* 9 1477–1487. 10.1210/mend.9.11.8584025 8584025

[B61] Love M. I. Huber W. Anders S. (2014). Moderated estimation of fold change and dispersion for RNA-seq data with DESeq2. *Genome Biol.* 15:550. 10.1186/s13059-014-0550-8 25516281 PMC4302049

[B62] Lu M. L. Schneider M. C. Zheng Y. Zhang X. Richie J. P. (2001). Caveolin-1 interacts with androgen receptor a positive modulator of androgen receptor mediated transactivation. *J. Biol. Chem.* 276 13442–13451. 10.1074/jbc.M006598200 11278309

[B63] Mak A. Kow N. Y. (2014). The pathology of T cells in systemic lupus erythematosus. *J. Immunol. Res.* 2014:419029. 10.1155/2014/419029 24864268 PMC4017881

[B64] Markou T. Cullingford T. E. Giraldo A. Weiss S. C. Alsafi A. Fuller S. J. (2008). Glycogen synthase kinases 3α and 3β in cardiac myocytes: regulation and consequences of their inhibition. *Cell. Signal.* 20 206–218. 10.1016/j.cellsig.2007.10.004 17993264

[B65] Miret C. Molina R. Filella X. García-Carrasco M. Claver G. Ingelmo M. (2003). Relationship of p53 with other oncogenes, cytokines and systemic lupus erythematosus activity. *TBI* 24 185–188. 10.1159/000074428 14654712

[B66] Navé B. T. Ouwens M. Withers D. J. Alessi D. R. Shepherd P. R. (1999). Mammalian target of rapamycin is a direct target for protein kinase B: identification of a convergence point for opposing effects of insulin and amino-acid deficiency on protein translation. *Biochem. J.* 344 427–431. 10567225 PMC1220660

[B67] Niculescu F. Nguyen P. Niculescu T. Rus H. Rus V. Via C. S. (2003). Pathogenic T cells in murine lupus exhibit spontaneous signaling activity through phosphatidylinositol 3-kinase and mitogen-activated protein kinase pathways. *Arthr. Rheum.* 48 1071–1079. 10.1002/art.10900 12687551

[B68] Nienhuis H. L. de Leeuw K. Bijzet J. Smit A. Schalkwijk C. G. Graaff R. (2008). Skin autofluorescence is increased in systemic lupus erythematosus but is not reflected by elevated plasma levels of advanced glycation endproducts. *Rheumatology (Oxford)* 47 1554–1558. 10.1093/rheumatology/ken302 18701539

[B69] Park J. K. Kim J.-Y. Moon J. Y. Ahn E. Y. Lee E. Y. Lee E. B. (2016). Altered lipoproteins in patients with systemic lupus erythematosus are associated with augmented oxidative stress: a potential role in atherosclerosis. *Arthr. Res. Ther.* 18:306. 10.1186/s13075-016-1204-x 28038677 PMC5203709

[B70] Perez-Diez A. Morgun A. Shulzhenko N. (2013). *Microarrays for Cancer Diagnosis and Classification. Landes Bioscience.* Available online at: https://www.ncbi.nlm.nih.gov/books/NBK6624/ (accessed July 19, 2019). 10.1007/978-0-387-39978-2_8 17265718

[B71] Pines A. Bivi N. Romanello M. Damante G. Kelley M. R. Adamson E. D. (2005). Cross-regulation between Egr-1 and APE/Ref-1 during early response to oxidative stress in the human osteoblastic HOBIT cell line: Evidence for an autoregulatory loop. *Free Radical Res.* 39 269–281. 10.1080/10715760400028423 15788231

[B72] Pons-Estel G. J. Alarcón G. S. Scofield L. Reinlib L. Cooper G. S. (2010). Understanding the epidemiology and progression of systemic lupus erythematosus. *Sem. Arthr. Rheum.* 39 257–268. 10.1016/j.semarthrit.2008.10.007 19136143 PMC2813992

[B73] Prokunina L. Alarcon-Riquelme M. (2004). The genetic basis of systemic lupus erythematosus—knowledge of today and thoughts for tomorrow. *Hum. Mol. Genet.* 13 R143–R148. 10.1093/hmg/ddh076 14764622

[B74] Rahman K. M. T. Islam M. F. Banik R. S. Honi U. Diba F. S. Sumi S. S. (2013). *Changes in Protein Interaction Networks Between Normal and Cancer Conditions: Total Chaos or Ordered Disorder? Network Biology.* Available online at: http://agris.fao.org/agris-search/search.do?recordID=CN2013200002 (accessed November 19, 2019).

[B75] Razani B. Lisanti M. P. (2001). Caveolins and caveolae: molecular and functional relationships. *Exp. Cell Res.* 271 36–44. 10.1006/excr.2001.5372 11697880

[B76] Recio J. A. Merlino G. (2003). Hepatocyte growth factor/scatter factor induces feedback up-regulation of CD44v6 in melanoma cells through Egr-1. *Cancer Res.* 63 1576–1582. 12670907

[B77] Rena G. Guo S. Cichy S. C. Unterman T. G. Cohen P. (1999). Phosphorylation of the transcription factor forkhead family member FKHR by protein kinase B. *J. Biol. Chem.* 274 17179–17183. 10.1074/jbc.274.24.17179 10358075

[B78] Ritchie M. E. Phipson B. Wu D. Hu Y. Law C. W. Shi W. (2015). limma powers differential expression analyses for RNA-sequencing and microarray studies. *Nucleic Acids Res.* 43:e47. 10.1093/nar/gkv007 25605792 PMC4402510

[B79] Robeva R. Tanev D. Andonova S. Kirilov G. Savov A. Stoycheva M. (2013). Androgen receptor (CAG)n polymorphism and androgen levels in women with systemic lupus erythematosus and healthy controls. *Rheumatol. Int.* 33 2031–2038. 10.1007/s00296-013-2687-2 23388696

[B80] Rui C. Li C. Xu W. Zhan Y. Li Y. Yang X. (2008). Involvement of Egr-1 in HGF-induced elevation of the human 5α-R1 gene in human hepatocellular carcinoma cells. *Biochem. J.* 411 379–386. 10.1042/BJ20071343 18215136

[B81] Russo G. Zegar C. Giordano A. (2003). Advantages and limitations of microarray technology in human cancer. *Oncogene* 22:6497. 10.1038/sj.onc.1206865 14528274

[B82] Sáenz-Corral I. Vega-Memíje M. E. Martínez-Luna E. Cuevas-González J. C. Rodríguez-Carreón A. A. de la Rosa J. J. B.-G. (2015). Apoptosis in chronic cutaneous lupus erythematosus, discoid lupus, and lupus profundus. *Int. J. Clin. Exp. Pathol.* 8 7260–7265. 26261624 PMC4525958

[B83] Salas T. R. Kim J. Vakar-Lopez F. Sabichi A. L. Troncoso P. Jenster G. (2004). Glycogen synthase kinase-3β is involved in the phosphorylation and suppression of androgen receptor activity. *J. Biol. Chem.* 279 19191–19200. 10.1074/jbc.M309560200 14985354

[B84] Santiago-Raber M.-L. Lawson B. R. Dummer W. Barnhouse M. Koundouris S. Wilson C. B. (2001). Role of cyclin kinase inhibitor p21 in systemic autoimmunity. *J. Immunol.* 167 4067–4074. 10.4049/jimmunol.167.7.4067 11564828

[B85] Sauer L. Gitenay D. Vo C. Baron V. T. (2010). Mutant p53 initiates a feedback loop that involves Egr-1/EGF receptor/ERK in prostate cancer cells. *Oncogene* 29 2628–2637. 10.1038/onc.2010.24 20190820 PMC2865566

[B86] Sekuliæ A. Hudson C. C. Homme J. L. Yin P. Otterness D. M. Karnitz L. M. (2000). A Direct linkage between the phosphoinositide 3-kinase-AKT signaling pathway and the mammalian target of rapamycin in mitogen-stimulated and transformed cells. *Cancer Res.* 60 3504–3513. 10910062

[B87] Shannon P. Markiel A. Ozier O. Baliga N. S. Wang J. T. Ramage D. (2003). Cytoscape: a software environment for integrated models of biomolecular interaction networks. *Genome Res.* 13 2498–2504. 10.1101/gr.1239303 14597658 PMC403769

[B88] Shen M. Yen A. (2008). c-Cbl Interacts with CD38 and promotes retinoic acid–induced differentiation and G0 arrest of human myeloblastic leukemia cells. *Cancer Res.* 68 8761–8769. 10.1158/0008-5472.CAN-08-1058 18974118 PMC4896297

[B89] Shin S. Y. Chin B. R. Lee Y. H. Kim J.-H. (2006). Involvement of glycogen synthase kinase-3β in hydrogen peroxide-induced suppression of Tcf/Lef-dependent transcriptional activity. *Cell. Signal.* 18 601–607. 10.1016/j.cellsig.2005.06.001 15993040

[B90] Shlomchik M. J. Craft J. E. Mamula M. J. (2001). From T to B and back again: positive feedback in systemic autoimmune disease. *Nat. Rev. Immunol.* 1 147–153. 10.1038/35100573 11905822

[B91] Siess W. Zangl K. J. Essler M. Bauer M. Brandl R. Corrinth C. (1999). Lysophosphatidic acid mediates the rapid activation of platelets and endothelial cells by mildly oxidized low density lipoprotein and accumulates in human atherosclerotic lesions. *Proc. Natl. Acad. Sci. U.S.A.* 96 6931–6936. 10359816 10.1073/pnas.96.12.6931 PMC22019

[B92] Skoura A. Hla T. (2009). Lysophospholipid receptors in vertebrate development, physiology, and pathology. *J. Lipid Res.* 50 S293–S298. 10.1194/jlr.R800047-JLR200 19065000 PMC2674736

[B93] Smith S. Fernando T. Wu P. W. Seo J. Ní Gabhann J. Piskareva O. (2017). MicroRNA-302d targets IRF9 to regulate the IFN-induced gene expression in SLE. *J. Autoimmu.* 79 105–111. 10.1016/j.jaut.2017.03.003 28318807

[B94] Smyth G. K. (2005). “limma: linear models for microarray data,” in *Bioinformatics and Computational Biology Solutions Using R and Bioconductor Statistics for Biology and Health*, eds Gentleman R. Carey V. J. Huber W. Irizarry R. A. Dudoit S. (New York, NY: Springer), 397–420. 10.1007/0-387-29362-0_23

[B95] Smyth S. S. Cheng H.-Y. Miriyala S. Panchatcharam M. Morris A. J. (2008). Roles of lysophosphatidic acid in cardiovascular physiology and disease. *Biochim. Biophys. Acta (BBA) Mol. Cell Biol. Lipids* 1781 563–570. 10.1016/j.bbalip.2008.05.008 18586114 PMC2572771

[B96] Szklarczyk D. Gable A. L. Lyon D. Junge A. Wyder S. Huerta-Cepas J. (2019). STRING v11: protein–protein association networks with increased coverage, supporting functional discovery in genome-wide experimental datasets. *Nucleic Acids Res.* 47 D607–D613. 10.1093/nar/gky1131 30476243 PMC6323986

[B97] Szklarczyk D. Morris J. H. Cook H. Kuhn M. Wyder S. Simonovic M. (2017). The STRING database in 2017: quality-controlled protein–protein association networks, made broadly accessible. *Nucleic Acids Res.* 45 D362–D368. 10.1093/nar/gkw937 27924014 PMC5210637

[B98] Tan E. M. Cohen A. S. Fries J. F. Masi A. T. Mcshane D. J. Rothfield N. F. (1982). The 1982 revised criteria for the classification of systemic lupus erythematosus. *Arthr. Rheum.* 25 1271–1277. 10.1002/art.1780251101 7138600

[B99] Tang E. D. Nuñez G. Barr F. G. Guan K.-L. (1999). Negative regulation of the forkhead transcription factor FKHR by Akt. *J. Biol. Chem.* 274 16741–16746. 10.1074/jbc.274.24.16741 10358014

[B100] Tang H. Tan G. Guo Q. Pang R. Zeng F. (2009). Abnormal activation of the Akt-GSK3β signaling pathway in peripheral blood T cells from patients with systemic lupus erythematosus. *Cell Cycle* 8 2789–2793. 10.4161/cc.8.17.9446 19652548

[B101] Thirumal Kumar D. Jain N. Evangeline J. Kamaraj B. Siva R. Zayed H. (2019). A computational approach for investigating the mutational landscape of RAC-alpha serine/threonine-protein kinase (AKT1) and screening inhibitors against the oncogenic E17K mutation causing breast cancer. *Comput. Biol. Med.* 115:103513. 10.1016/j.compbiomed.2019.103513 31698236

[B102] Tsokos G. C. Lo M. S. Reis P. C. Sullivan K. E. (2016). New insights into the immunopathogenesis of systemic lupus erythematosus. *Nat. Rev. Rheumatol.* 12 716–730. 10.1038/nrrheum.2016.186 27872476

[B103] Tzivion G. Dobson M. Ramakrishnan G. (2011). FoxO transcription factors; Regulation by AKT and 14-3-3 proteins. *Biochim. Biophy. Acta (BBA) Mol. Cell Res.* 1813 1938–1945. 10.1016/j.bbamcr.2011.06.002 21708191

[B104] Veeranki S. Choubey D. (2010). Systemic lupus erythematosus and increased risk to develop B cell malignancies: role of the p200-family proteins. *Immunol. Lett.* 133 1–5. 10.1016/j.imlet.2010.06.008 20599558 PMC2926135

[B105] Vences-Catalán F. Rajapaksa R. Levy S. Santos−Argumedo L. (2012). The CD19/CD81 complex physically interacts with CD38 but is not required to induce proliferation in mouse B lymphocytes. *Immunology* 137 48–55. 10.1111/j.1365-2567.2012.03602.x 22564057 PMC3449246

[B106] Wang J. Liu Y. Zhao J. Xu J. Li S. Qin X. (2017). P-glycoprotein gene MDR1 polymorphisms and susceptibility to systemic lupus erythematosus in Guangxi population: a case-control study. *Rheumatol. Int.* 37 537–545. 10.1007/s00296-017-3652-2 28154898

[B107] Wolfrum C. Besser D. Luca E. Stoffel M. (2003). Insulin regulates the activity of forkhead transcription factor Hnf-3β/Foxa-2 by Akt-mediated phosphorylation and nuclear/cytosolic localization. *Proc. Natl. Acad. Sci. U.S.A.* 100 11624–11629. 10.1073/pnas.1931483100 14500912 PMC208808

[B108] Yamauchi A. Bloom E. T. (1997). Control of cell cycle progression in human natural killer cells through redox regulation of expression and phosphorylation of retinoblastoma gene product protein. *Blood* 89 4092–4099. 10.1182/blood.V89.11.4092 9166850

[B109] Yang L. V. Radu C. G. Wang L. Riedinger M. Witte O. N. (2005). Gi-independent macrophage chemotaxis to lysophosphatidylcholine via the immunoregulatory GPCR G2A. *Blood* 105 1127–1134. 10.1182/blood-2004-05-1916 15383458

[B110] Yang W. Lau Y. L. (2015). Solving the genetic puzzle of systemic lupus erythematosus. *Pediatr. Nephrol.* 30 1735–1748. 10.1007/s00467-014-2947-8 25239301

[B111] Yeh P. Y. Kuo S.-H. Yeh K.-H. Chuang S.-E. Hsu C.-H. Chang W. C. (2006). A pathway for tumor necrosis factor-α-induced Bcl10 nuclear translocation Bcl10 is up-regulated by nf-κb and phosphorylated by Akt1 and then complexes with bcl3 to enter the nucleus. *J. Biol. Chem.* 281 167–175. 10.1074/jbc.M511014200 16280327

[B112] Yu J. Akishita M. Eto M. Koizumi H. Hashimoto R. Ogawa S. (2012). Src kinase-mediates androgen receptor-dependent non-genomic activation of signaling cascade leading to endothelial nitric oxide synthase. *Biochem. Biophys. Res. Commun.* 424 538–543. 10.1016/j.bbrc.2012.06.151 22771325

[B113] Zhou Y. Yuan J. Pan Y. Fei Y. Qiu X. Hu N. (2009). T cell CD40LG gene expression and the production of IgG by autologous B cells in systemic lupus erythematosus. *Clin. Immunol.* 132 362–370. 10.1016/j.clim.2009.05.011 19520616 PMC2810511

[B114] Zhu H. Luo H. Yan M. Zuo X. Li Q.-Z. (2015). Autoantigen microarray for high-throughput autoantibody profiling in systemic lupus erythematosus. *Genom. Proteom. Bioinformatics* 13 210–218. 10.1016/j.gpb.2015.09.001 26415621 PMC4610965

