# Supplementary material for: Dysregulation of Signaling Pathways Due to Differentially Expressed Genes From the B-Cell Transcriptomes of Systemic Lupus Erythematosus Patients – A Bioinformatics Approach
Source: Front Bioeng Biotechnol. 2020 Apr 30;8:276. doi: 10.3389/fbioe.2020.00276 (PMC7203449; doi:10.3389/fbioe.2020.00276)
Supplement: Supplementary file 1 [file Table_1.docx]

**Supplementary Table 1. Genes that are differentially expressed between the case and control of GSE30153 dataset are tabulated.**

| Gene symbol | log2FC | p-value | Gene symbol | log2FC | p-value |
| --- | --- | --- | --- | --- | --- |
| *VEGFA* | -0.347 | 0.0000916 | *SIGLEC5* | 0.423 | 0.0039703 |
| *HNRNPLL* | 0.318 | 0.0001869 | *SMDT1* | 0.545 | 0.003972 |
| *TRAF3IP2* | 0.37 | 0.0001897 | *ZMAT3* | 0.457 | 0.0041134 |
| *CD24* | 0.462 | 0.0001954 | *CAMK2N1* | 0.735 | 0.004128 |
| *NAPSB* | 0.343 | 0.0002543 | *PRKCE* | 0.528 | 0.0041866 |
| *CCDC92* | 0.387 | 0.0002612 | *PDIA4* | -1.014 | 0.0041988 |
| *ZCCHC14* | 0.39 | 0.0002706 | *ABI2* | 0.375 | 0.0042001 |
| *ST14* | 0.613 | 0.0002924 | *GATAD2A* | 0.252 | 0.0042161 |
| *GAB1* | -0.934 | 0.000396 | *MPEG1* | 0.589 | 0.0042454 |
| *MOXD1* | -0.95 | 0.0004291 | *BCOR* | 0.479 | 0.0042462 |
| *NUFIP2* | 0.264 | 0.000483 | *DCLRE1C* | 0.376 | 0.0042463 |
| *GNG11* | 0.584 | 0.0004954 | *NT5DC1* | 0.271 | 0.0043132 |
| *CYTIP* | -0.45 | 0.0005006 | *SPATS2L* | -0.232 | 0.0043327 |
| *IGLJ3** | -0.733 | 0.0005034 | *CHAC2* | -1.071 | 0.004354 |
| *CD1C* | 1.068 | 0.0005281 | *SMAGP* | 0.464 | 0.0043636 |
| *PMEPA1* | 0.8 | 0.0006012 | *ARHGAP24* | 0.964 | 0.0044 |
| *CARNS1* | 0.516 | 0.0006111 | *IL6ST* | -0.511 | 0.0044137 |
| *RHOBTB3** | 0.551 | 0.0007008 | *MAN1A1* | -1.111 | 0.004425 |
| *MOXD1** | -0.718 | 0.0007088 | *LPGAT1* | 0.469 | 0.0044317 |
| *IKZF3* | 0.287 | 0.0007292 | *IL6ST** | -0.486 | 0.004534 |
| *EGR1** | 1.22 | 0.0007385 | *CHEK1* | -0.273 | 0.0045775 |
| *TLR10* | 0.495 | 0.0007504 | *ST14** | 0.596 | 0.0046236 |
| *MTMR3* | 0.506 | 0.0007562 | *IGLV1-44* | -0.845 | 0.0046571 |
| *CD84* | 0.342 | 0.0008013 | *LINC00173* | -0.328 | 0.004725 |
| *IL7* | 0.745 | 0.0008393 | *CREM* | -0.236 | 0.0047298 |
| *RPL15* | 0.421 | 0.0008805 | *SLC25A37* | 0.395 | 0.0047473 |
| *ZBTB42* | 0.309 | 0.0009025 | *HIVEP2* | -0.155 | 0.0047485 |
| *IGHM** | -0.617 | 0.0009193 | *PGS1* | 0.166 | 0.0047991 |
| *WWC2-AS2* | 0.313 | 0.0009656 | *CAV1* | -1.516 | 0.0048324 |
| *GPM6A* | 1.052 | 0.0009745 | *LFNG* | 0.34 | 0.0048345 |
| *AKT1* | 0.294 | 0.0010146 | *TYMS** | -0.697 | 0.0048969 |
| *C22orf34* | 0.481 | 0.0010166 | *E2F7* | -0.359 | 0.0049136 |
| *MFSD12* | 0.257 | 0.0010403 | *FAM126A* | -0.548 | 0.0049271 |
| *CCDC58* | 0.481 | 0.0011389 | *ZDHHC14* | 0.391 | 0.0049752 |
| *PATZ1* | 0.399 | 0.0011683 | *STT3B* | -0.392 | 0.0049784 |
| *IGK** | -0.765 | 0.0011697 | *NXPE3* | -0.514 | 0.0049838 |
| *MRPL10* | -0.201 | 0.0011889 | *SAR1B* | -1.176 | 0.0049953 |
| *ZNF35* | -0.235 | 0.0012128 | *TCF3* | -0.388 | 0.0049971 |
| *GAB1** | -0.702 | 0.0012628 | *SUMF1* | 0.298 | 0.0050142 |
| *MGC16275* | 0.243 | 0.0013362 | *ERLEC1* | -0.811 | 0.005027 |
| *TRIM2* | 0.481 | 0.0013643 | *E2F6* | -0.272 | 0.0050504 |
| *IGK* | -0.902 | 0.0013825 | *IGLJ3** | -0.615 | 0.0050587 |
| *ARMC2* | -0.223 | 0.0013906 | *BMP6* | -0.38 | 0.0050596 |
| *AQP3* | -0.49 | 0.0014031 | *CUX1* | 0.301 | 0.0051064 |
| *TOP1MT* | 0.505 | 0.0014342 | *LDOC1L* | 0.307 | 0.0051433 |
| *MLIP* | -0.742 | 0.0014912 | *IGK** | -0.338 | 0.0052097 |
| *SORD* | 0.422 | 0.0015796 | *IGLJ3** | -0.661 | 0.0052223 |
| *SNN* | 0.453 | 0.001631 | *RNF141* | 0.505 | 0.0052276 |
| *KIAA2022* | 0.446 | 0.0016475 | *PTPRJ* | 0.483 | 0.0052933 |
| *RGP1* | 0.322 | 0.0016558 | *ERAP1* | -1.047 | 0.005321 |
| *C10orf128* | 0.904 | 0.0016968 | *IGLL3P* | -0.402 | 0.0053528 |
| *IGK* | -0.717 | 0.0017204 | *OVCA2* | 0.241 | 0.0053767 |
| *PROX1* | -0.185 | 0.0017533 | *CHML* | 0.244 | 0.0053815 |
| *IGKV1OR2-118* | -0.804 | 0.001766 | *TRAM1* | -0.283 | 0.0053872 |
| *C20orf196* | 0.169 | 0.0017772 | *SMG7* | -0.241 | 0.0053983 |
| *ING1** | 0.273 | 0.0018099 | *MTFR1* | -0.394 | 0.0054338 |
| *EFCAB13* | 0.222 | 0.0018233 | *SLC25A51* | -0.277 | 0.0054397 |
| *IGKV1OR2-108* | -0.772 | 0.0018794 | *TXNDC15* | -0.692 | 0.0054783 |
| *LTBP3* | 0.511 | 0.001915 | *IFI27L1* | -0.606 | 0.0054811 |
| *ABHD15* | 0.413 | 0.0019828 | *TNFSF9* | 0.191 | 0.0054848 |
| *PTPN18* | 0.31 | 0.0019967 | *OSBPL10* | 0.432 | 0.005507 |
| *RPL35A* | 0.503 | 0.0020216 | *TFDP2* | -0.321 | 0.0055087 |
| *ARHGAP35* | 0.379 | 0.0022859 | *ZNF395* | 0.4 | 0.0055284 |
| *ING1* | 0.302 | 0.0022904 | *SGPP2* | -0.322 | 0.0055287 |
| *TPM1* | 0.277 | 0.0023085 | *CLMN* | 0.522 | 0.0055679 |
| *USP32* | 0.18 | 0.002324 | *IGLJ3* | -0.963 | 0.0055842 |
| *JCHAIN* | -0.754 | 0.002389 | *FCHO2* | 0.529 | 0.0056374 |
| *GNS* | -0.303 | 0.0024102 | *SLC2A5* | -0.327 | 0.0056649 |
| *EIF4EBP2* | 0.275 | 0.0024122 | *HADH* | 0.277 | 0.0057291 |
| *RAB1A* | -0.386 | 0.0025135 | *FDFT1* | 0.432 | 0.0057804 |
| *FKBP11* | -0.736 | 0.0025445 | *OSBP* | -0.4 | 0.0058067 |
| *TIMM23B* | -0.375 | 0.0025985 | *ARF4* | -1.044 | 0.0058274 |
| *IGHM* | -0.665 | 0.0026007 | *ACOX1* | -0.372 | 0.0058504 |
| *SUB1* | -0.418 | 0.0026334 | *PRUNE1* | 0.262 | 0.005869 |
| *NAPSB** | 0.334 | 0.0026486 | *SSMEM1* | 0.166 | 0.0059381 |
| *MIR7110* | -1.4 | 0.0027212 | *PPIB* | -0.671 | 0.0060105 |
| *RRM2* | -2.406 | 0.0027527 | *GALNT2* | -0.527 | 0.0060122 |
| *MGLL* | -0.556 | 0.0027929 | *ITGAX* | -0.413 | 0.006013 |
| *PAM* | -0.282 | 0.0028015 | *PHF19* | -0.264 | 0.0060234 |
| *PRDX4* | -0.989 | 0.0028118 | *CKAP2* | -0.628 | 0.0060634 |
| *MARCKS* | 0.645 | 0.0028229 | *CLMN** | 0.441 | 0.0061044 |
| *UNC5B* | -0.188 | 0.0028326 | *IGK** | -0.212 | 0.0061066 |
| *PARP14* | -0.246 | 0.0028492 | *ZNF559* | 0.4 | 0.006144 |
| *IGK** | -0.247 | 0.0028565 | *FOXP1* | 0.278 | 0.0061453 |
| *IGKC* | -0.728 | 0.0028859 | *IGK** | -0.834 | 0.0061504 |
| *SAR1A* | -0.981 | 0.0028971 | *NCF4* | 0.305 | 0.0061594 |
| *DSE* | 1.125 | 0.0029131 | *MAN1A2* | -0.472 | 0.0061739 |
| *ZNF747* | -0.218 | 0.0029499 | *MAPK9* | 0.246 | 0.0061742 |
| *GPM6A** | 1.043 | 0.002981 | *LGALS14* | -0.244 | 0.0061944 |
| *RGS13* | -0.607 | 0.0029892 | *SPECC1L* | 0.253 | 0.0062091 |
| *RRM2** | -2.152 | 0.0030096 | *DDAH2* | 0.338 | 0.0062227 |
| *GAS6* | -0.482 | 0.0030307 | *SRGN* | -0.757 | 0.0062288 |
| *ARHGAP24** | 0.84 | 0.003079 | *SH3GLB1* | -0.4 | 0.0062758 |
| *KNL1* | -0.238 | 0.0030816 | *SLC35F6* | -0.231 | 0.0063156 |
| *RPA1* | 0.288 | 0.0030886 | *CYTH4* | 0.272 | 0.0063264 |
| *ARHGAP24** | 0.757 | 0.0031128 | *BMS1P20* | -0.953 | 0.0063532 |
| *BRD3* | 0.35 | 0.0031386 | *TP53I13* | 0.31 | 0.0063622 |
| *IGHM** | -0.493 | 0.0031467 | *YME1L1* | -0.336 | 0.0063952 |
| *SIGLEC10* | 0.484 | 0.0031591 | *SLC44A1* | -1.219 | 0.0035298 |
| *CD38* | -1.702 | 0.0031747 | *KIF13A* | 0.707 | 0.0035451 |
| *TYMS* | -1.923 | 0.0032032 | *CDK6* | -0.525 | 0.0035874 |
| *KIAA1143* | 0.34 | 0.0032401 | *GAB1** | -0.73 | 0.003617 |
| *EGR1* | 1.034 | 0.0032807 | *WDFY4* | 0.339 | 0.0038117 |
| *SCARB2* | -0.509 | 0.0033268 | *SPIB* | 0.481 | 0.0038344 |
| *CUL3* | 0.709 | 0.0033399 | *PTPN6* | 0.349 | 0.0038475 |
| *MAN2B2* | 0.287 | 0.0033422 | *MAN1A1** | -0.933 | 0.0038783 |
| *ERP44* | -0.312 | 0.0033701 | *POLA2* | 0.225 | 0.0038969 |
| *RHOBTB3* | 0.554 | 0.0034143 | *MRRF* | -0.222 | 0.0039078 |
| *BTBD2* | 0.233 | 0.0034511 | *TSN* | 0.336 | 0.003956 |
| *NDFIP2* | -0.334 | 0.003484 | *SPIB** | 0.291 | 0.0039692 |
| *SUB1** | -0.409 | 0.0034884 | *PDK3* | 0.363 | 0.0035245 |
| *FYN* | 0.382 | 0.0034943 | *ELL2* | -1.354 | 0.0035256 |
| *SLC43A2* | 0.294 | 0.0035059 | *PRKCB* | 0.238 | 0.0035259 |

**^* The asterisk denotes the DEGs with two or more different probes from the dataset. Significant DEGs were considered based on the log2FC (≥ 1 or ≤ -1) and/or p-value (< 0.05) and examined for further analysis.^**
